# Supplementary material for: Plasma lipidome dysregulation in frontotemporal dementia reveals shared, genotype‐specific, and severity‐linked alterations
Source: Alzheimers Dement. 2025 Sep 8;21(9):e70631. doi: 10.1002/alz.70631 (PMC12417310; doi:10.1002/alz.70631)
Supplement: Supplementary file 1 — Supporting Information [file ALZ-21-e70631-s005.pdf]

Supp Figure 1

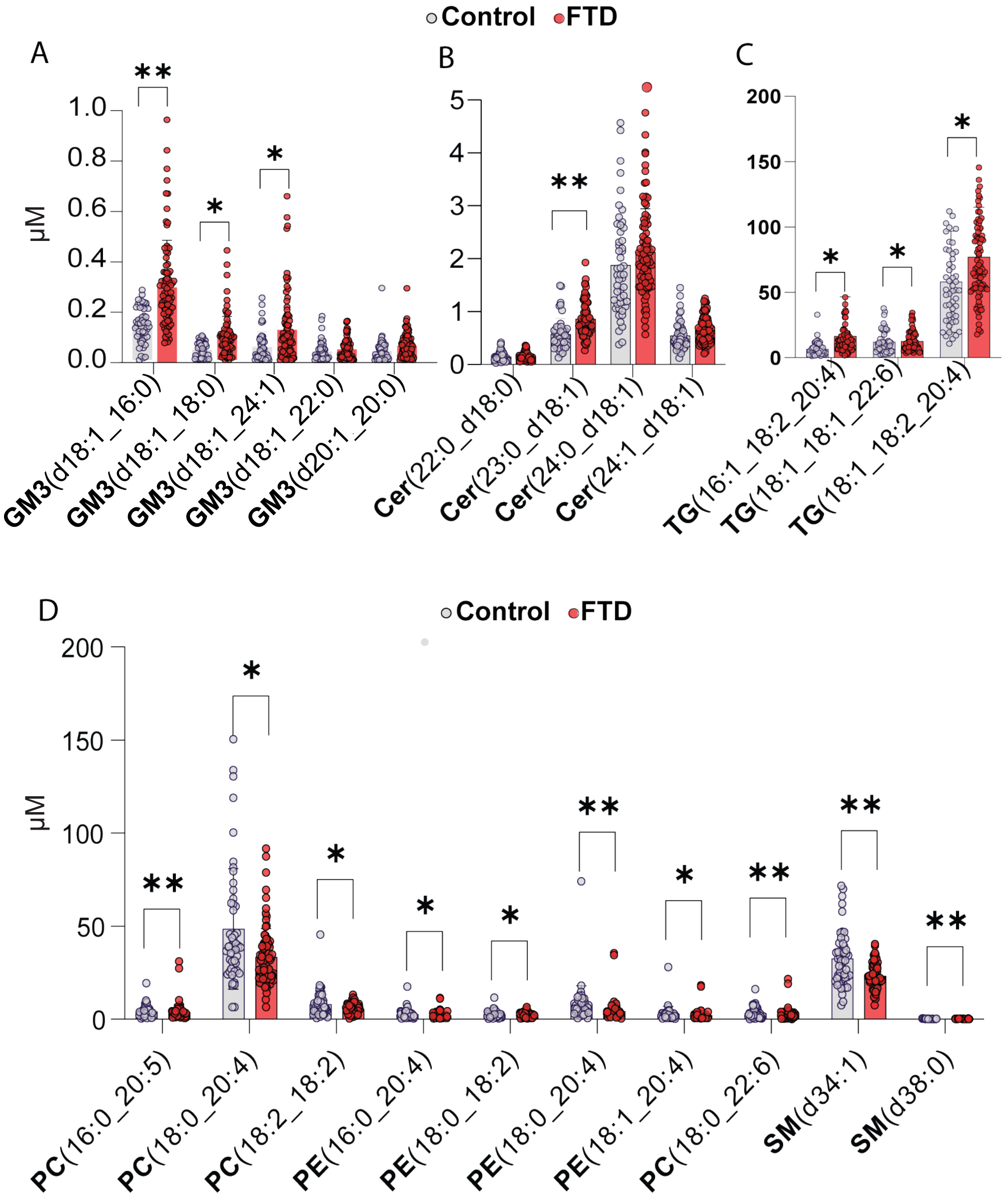

Supplementary Figure 1. Alteration of plasma lipid species in FTD, compared to controls. (**A-C**) Levels of specific plasma ganglioside species (A), certain ceramide species (B), and certain TG species were significantly higher in FTD cases than controls. (**D**) Levels of specific plasma phospholipid and sphingomyelin species were lower in FTD cases than controls. Bars represent the mean ± standard deviation (SD). Each dot represents to samples. Data are presented as mean ± SD, *p<0.05, **p<0.01 (multiple parametric tests, with Welch t-test).
