# Supplementary material for: Plasma lipidome dysregulation in frontotemporal dementia reveals shared, genotype‐specific, and severity‐linked alterations
Source: Alzheimers Dement. 2025 Sep 8;21(9):e70631. doi: 10.1002/alz.70631 (PMC12417310; doi:10.1002/alz.70631)
Supplement: Supplementary file 2 — Supporting Information [file ALZ-21-e70631-s004.pdf]

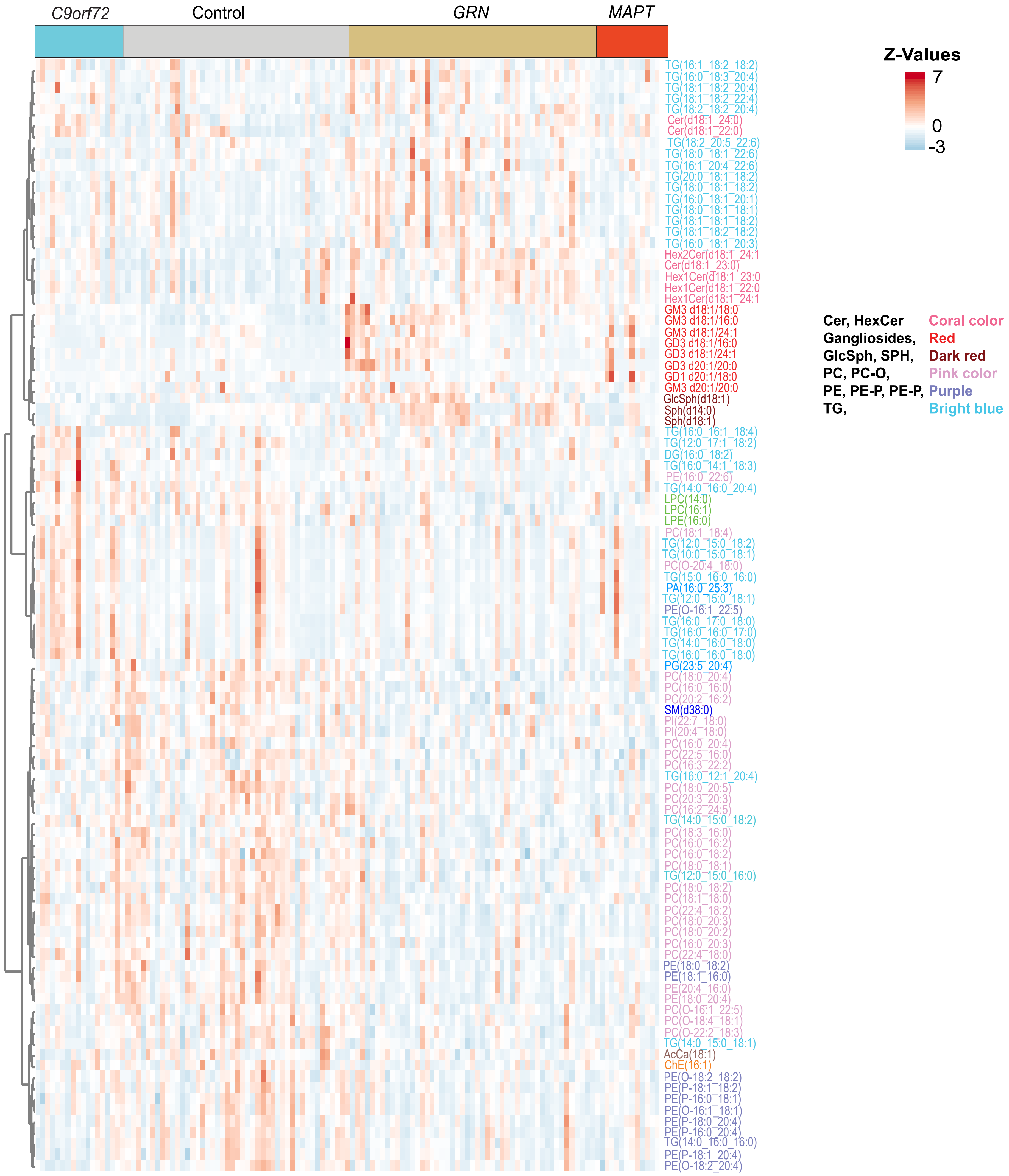

Supplementary Figure 2. Unsupervised heatmap displaying the top 150 lipid species with the greatest magnitude of change across control, FTD-*GRN*, FTD-*MAPT*, and FTD-*C9orf72* groups. Lipid classes include ceramides (dark red), gangliosides (golden yellow), phospholipids (sky blue, dark green), and triacylglycerols (red). Z-score normalization highlights distinct lipid alterations associated with different conditions.
